# An Insight into Clinicopathological Parameters and Prognostic Significance of Double Expressor Diffuse Large B-Cell Lymphoma in a Tertiary Care Center

**DOI:** 10.7759/cureus.97806

**Published:** 2025-11-25

**Authors:** Ramya Lakshminarayanan, Nagarajan Priyathersini, Sri Gayathri Shanmugam, Arthi Mohanendran, Viha Vaishalee Moganavalli Giridhar

**Affiliations:** 1 Pathology, Sri Ramachandra Institute of Higher Education and Research, Chennai, IND

**Keywords:** dlbcl-nos, double expressor diffuse large b-cell lymphoma (de-dlbcl), gcb, hans algorithm, non-gcb

## Abstract

Introduction

Diffuse large B-cell lymphoma (DLBCL) is the most common subtype of non-Hodgkin lymphoma (NHL). A distinct, high-risk subset-double expressor DLBCL shows immunohistochemical (IHC) co-expression of BCL2 and c-MYC.

Objective

To evaluate the clinicopathological spectrum and prognostic significance of double expressor DLBCL.

Methods

This retrospective study included 49 patients diagnosed with DLBCL over a four-year period at a tertiary care center. IHC analysis using a non-Hodgkin lymphoma panel was performed, and subtyping was done using the Hans algorithm to classify cases as germinal center B-cell-like (GCB) or non-germinal center B. Double expressor status was defined by IHC expression thresholds for BCL2 and c-MYC.

Results

Of the 49 cases, 27 (55.1%) were GCB and 22 (44.8%) were non-GCB. Twenty-three patients (46.9%) met criteria for double expressor DLBCL-11 GCB (47.8%) and 12 non-GCB (52.2%). Follow-up data from 27 patients revealed a significantly higher early mortality rate among double expressor cases, particularly within the first six months, suggesting an unfavorable prognosis. Histopathology showed diffuse effacement of lymph node architecture with proliferation of medium- to large-sized atypical lymphoid cells.

Conclusion

Double expressor DLBCL represents a clinically aggressive variant with a distinctly worse prognosis than DLBCL-not otherwise specified (NOS). Double expressor DLBCL poses a significant prognostic challenge within DLBCL-NOS and must be recognized as a key determinant in patient stratification and management.

## Introduction

Lymphoma is a malignant disease that arises from lymphocytes within lymphoid tissue. It is broadly classified into Hodgkin lymphoma and non-Hodgkin lymphoma. Non-Hodgkin lymphoma (NHL) constitutes more than 85% of all lymphoma cases. Among these, over 90% arise from mature B cells, with diffuse large B-cell lymphoma (DLBCL) being the most common subtype, accounting for approximately 24% of cases [1].

DLBCL not otherwise specified (NOS) is defined by its characteristic morphology and mature B-cell immunophenotype. It represents a heterogeneous group of tumors that vary in terms of their cell of origin, based on immunophenotypic markers, and exhibit diverse morphological, molecular, and genetic subtypes. Morphologically, DLBCL NOS consists of medium-sized to large neoplastic B cells with a diffuse growth pattern.

The classification of DLBCL based on the cell of origin is commonly done using the Hans algorithm [2,3], an immunohistochemistry-based method. According to this algorithm, the germinal center B-cell-like (GCB) subtype is identified by CD10 positivity, with or without BCL6 expression, and absence of MUM1. Alternatively, cases that are CD10-negative but BCL6-positive and MUM1-negative are also classified as GCB. The activated B-cell-like (ABC) subtype is characterized by CD10 negativity, with either BCL6 and MUM1 positivity or BCL6 negativity.

In addition to the cell of origin, the expression of oncogenic proteins such as c-MYC and BCL2 carries prognostic significance. DLBCL cases showing immunohistochemical (IHC) co-expression of c-MYC (≥40%) and BCL2 (≥50%) are classified as having double expressor [4]. This article has been presented as an E-paper presentation at TAPCON 2024.

## Materials and methods

This is a type of retrospective study, which is conducted at a tertiary care center, for a period of four years and six months from April 2020 to September 2024. The sample size for the study was 49. Data were collected from electronic medical records in the Department of Pathology. Cases included newly diagnosed and follow-up from previous diagnoses. A consecutive sampling technique was used for the study. Inclusion criteria included all DLBCL cases for the above period for which H&E and IHC slides are available. Exclusion criteria include DLBCL cases in the above period for which IHC slides are not available. IHC panel (CD45, CD10, CD3, CD5, CD20, BCL6, BCL2, c-MYC, MUM1, and Ki-67) was done for the classification of lymphomas and subcategorized based on cell of origin by HANS classification into germinal center B and non-germinal center B types. WHO classification of hematolymphoid tumors (2022) [4] recommends a cut-off value of 40% for IHC c-MYC expression and 50% for BCL2 protein expression for the double expressor subtype. The double expressor frequency with the association of cell of origin and clinical parameters, such as age, sex, Ki-67 proliferation index, site of origin (nodal or extranodal) of the patient, is analyzed with prognostic factors of DLBCL (NOS) with double expressor DLBCL.

Data entry was done using Microsoft Excel (Microsoft Corporation, Redmond, WA, USA), and analysis was done using the Statistical Package for the Social Sciences (SPSS) software version 26 (IBM Corp., Armonk, NY, USA). Descriptive statistics were calculated for background characteristics to summarize demographic and clinical variables. Categorical variables were analyzed, and the association was done using the chi-square test. A p-value of less than 0.05 was considered statistically significant.

## Results

Demographic and clinicopathological distribution of diffuse large B-cell lymphoma (not otherwise specified) cases in the study cohort

The study was conducted in a total of 49 cases, with the majority of cases occurring in males (63.3%) and individuals aged 50 years or older (77.5%). The germinal center B-cell (GCB) subtype was the most common (55.1%), with lymph nodes being the predominant site of origin (Table 1). Statistical test used for calculating the p-value by the chi-square test.

**Table 1:** Statistics of DLBCL NOS on study population(n=49). DLBCL: diffuse large B-cell lymphoma; NOS: not otherwise specified; GCB: germinal center B-cell.

Classification	Details	Frequency
By age group		
	Age less than 50 years	11 (22.4%)
	Age 50 and more	38 (77.5%)
By gender		
	Male	31 (63.3%)
	Female	18 (36.7%)
By site of origin		
	Nodal	26 (53.06%)
	Extranodal	23 (46.9%)
By subtype of DLBCL		
	GCB	27 (55.1%)
	Non-GCB	22 (44.8%)
By Ki-67 proliferation index (n=38)		
	<80	31 (81.6%)
	>80	7 (18.4%)

Statistics of double expressor

Among double expressor DLBCL cases, the majority, 73.9% were over 50 years of age, with 26.1% under 50. Males comprised 57% of this subgroup, while females accounted for 43%. Based on the Hans algorithm, 52.2% belonged to the non-GCB subtype and 47.8% to the GCB subtype with a P-value of 0.528. The Ki-67 proliferation index exceeded 80% in 35.3% of cases, whereas 64.7% showed lower proliferative activity with a p-value of 0.016, which is statistically significant (Table 2).

**Table 2:** Statistics of double expressor DLBCL on study population (n=49). DLBCL: diffuse large B-cell lymphoma; NOS: not otherwise specified; GCB: germinal center B-cell.

Classification	Details	Positive	Negative	Chi-square value	Degree of freedom	p-value
Age group						
	Age less than 50 years	6 (26.1%)	5 (19.2%)	0.330	1	0.566
	Age 50 and more	17 (73.9%)	21 (80.8%)			
Gender						
	Female	10 (43%)	8 (31%)	0.848	1	0.357
	Male	13 (57%)	18 (69%)			
Site of origin						
	Nodal	13 (59.1%)	10 (48.2%)	1.394	1	0.223
	Extranodal	9 (40.9%)	14 (51.8%)			
Subtype of DLBCL						
	GCB	11 (47.8%)	16 (61.5%)	0.399	1	0.528
	Non-GCB	12 (52.2%)	10 (38.5%)			
Ki-67 proliferation index						
	<80	11 (64.7%)	20 (95.2)	5.828	1	0.016 (significant)
	>80	6 (35.3%)	1 (4.8)			

Diffuse large B-cell lymphoma subtype 

**Figure 1:**
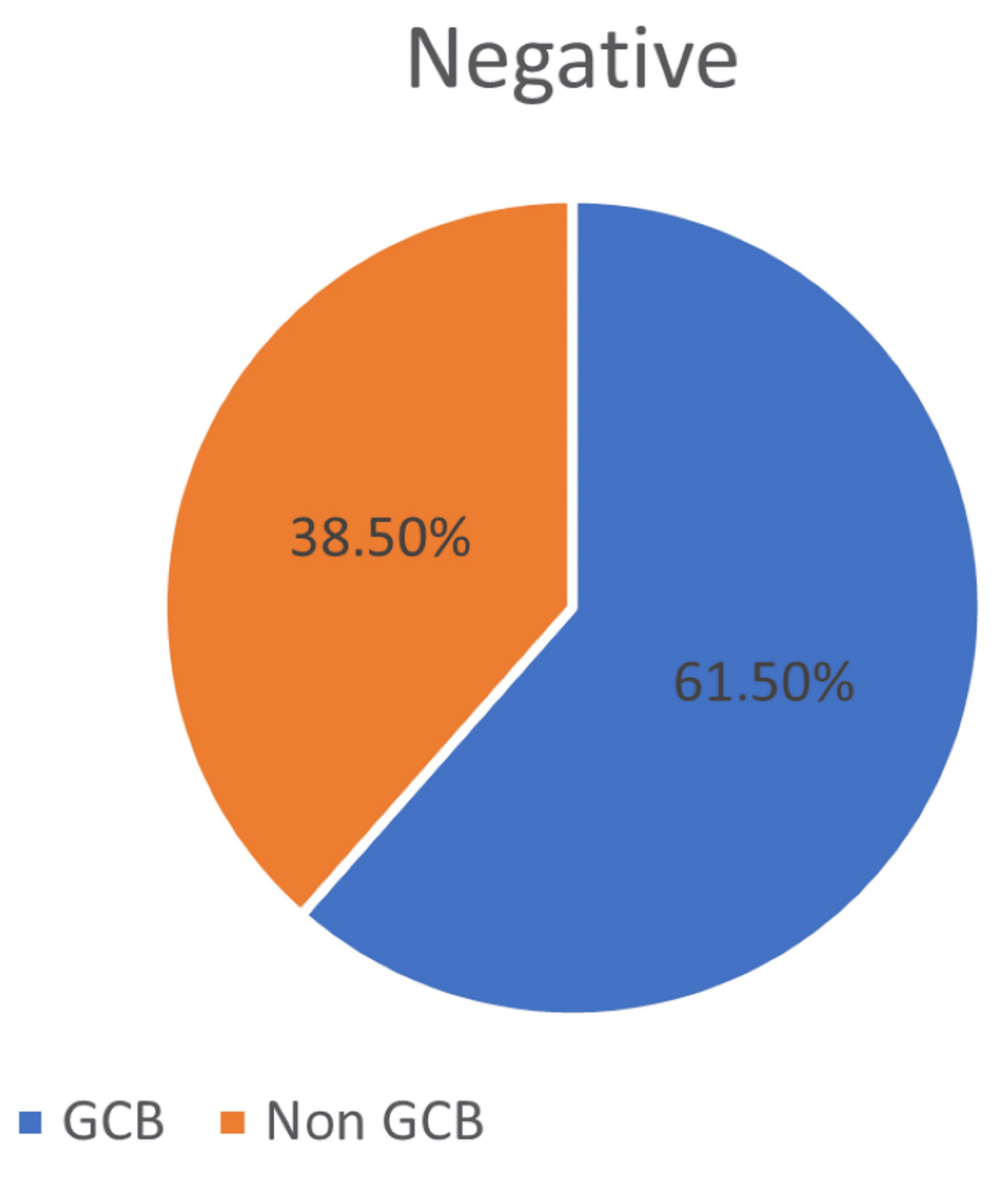
DLBCL subtype of DLBCL NOS. DLBCL: diffuse large B-cell lymphoma; NOS: not otherwise specified; GCB: germinal center B-cell.

Our cohort has 61.5% of GCB subtype and 38.5% of non-GCB subtype in DLBCL NOS (Figure 1).

Subtypes of double expressor

**Figure 2:**
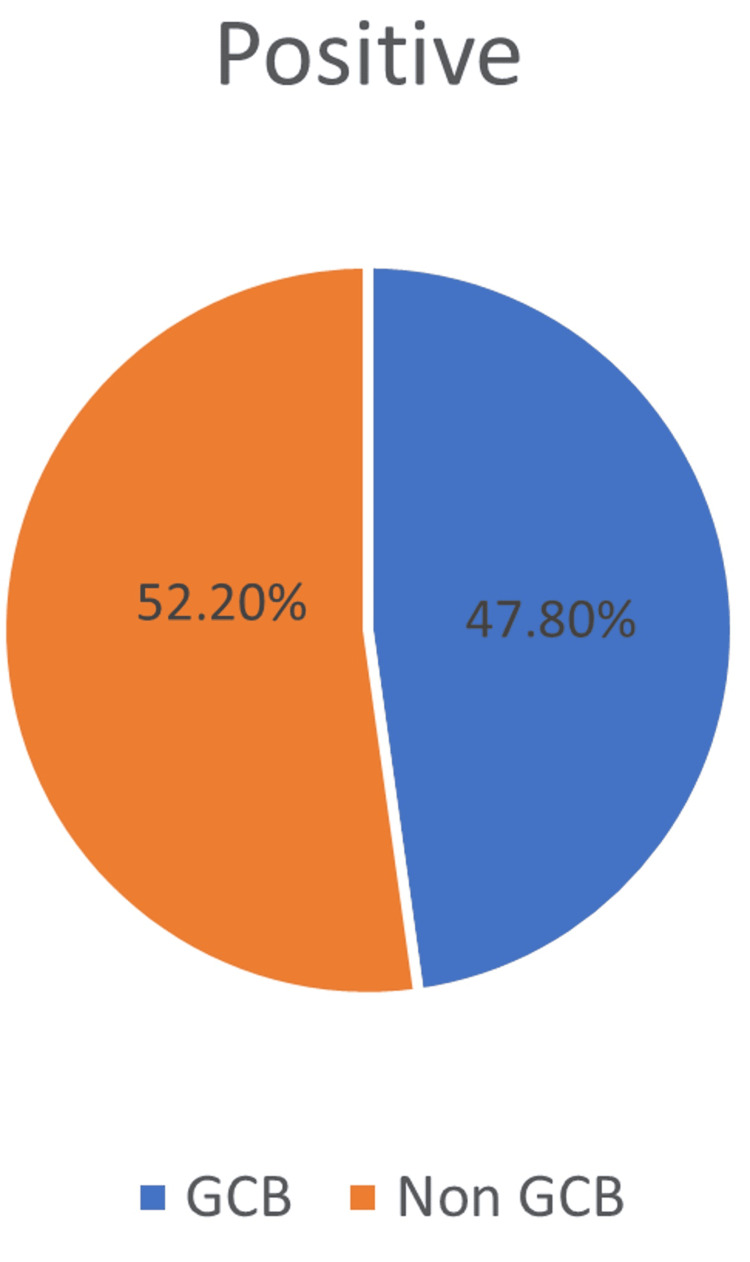
Subtype of double expressor. GCB: germinal center B-cell.

Our cohort has twelve cases (52.2%) of non-GCB subtype and eleven cases (47.8%) of GCB subtype in double expressor DLBCL (Figure 2).

Ki-proliferation index 

**Figure 3:**
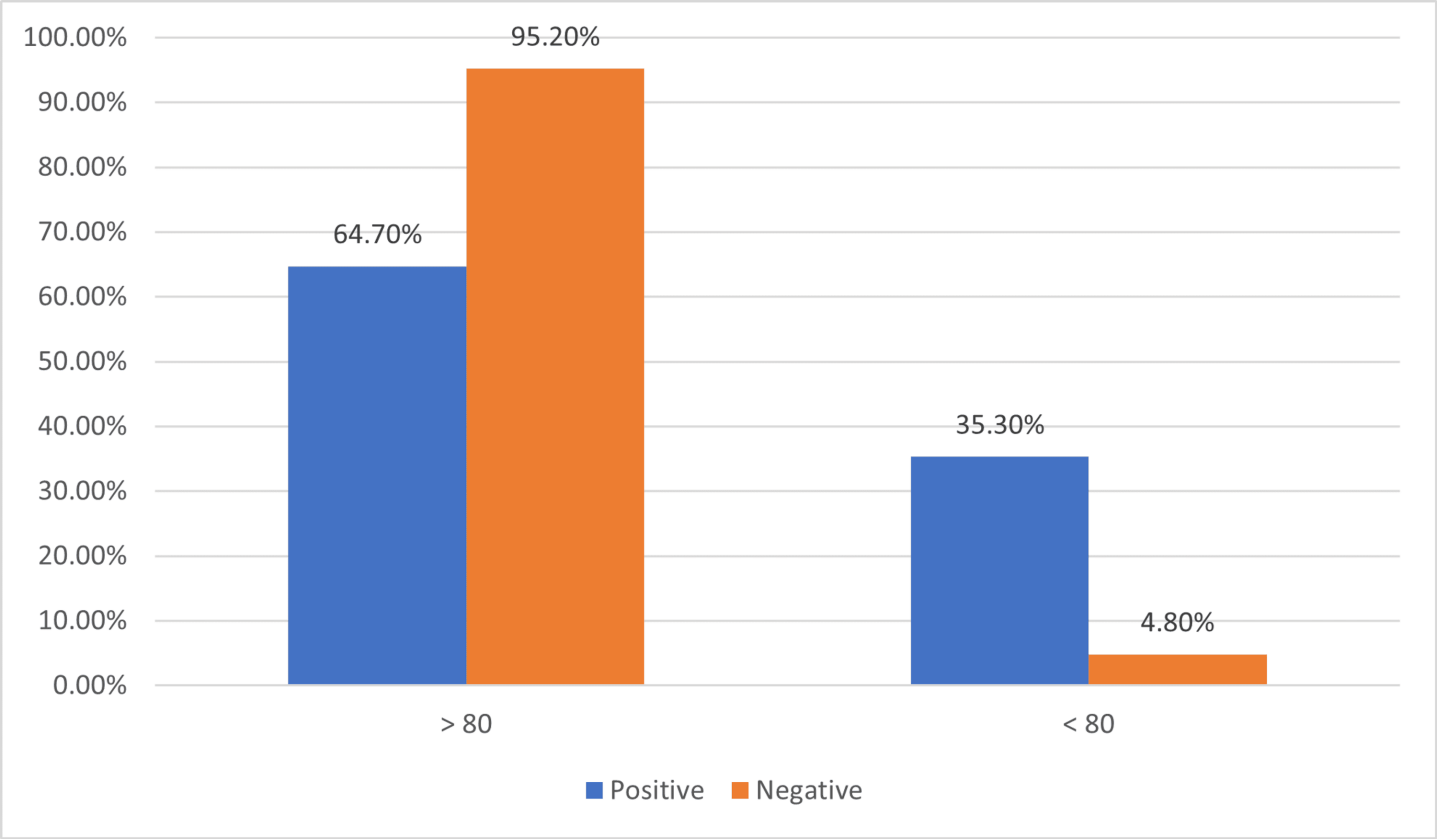
Ki-67 proliferation index.

In case of double expressor DLBCL, seventeen cases were evaluated, with six cases (35.3%) having a Ki-67 labeling index greater than 80% and 11 cases (64.7%) having a Ki-67 labeling index less than 80% (Figure 3).

Prognosis of double expressor 

**Figure 4:**
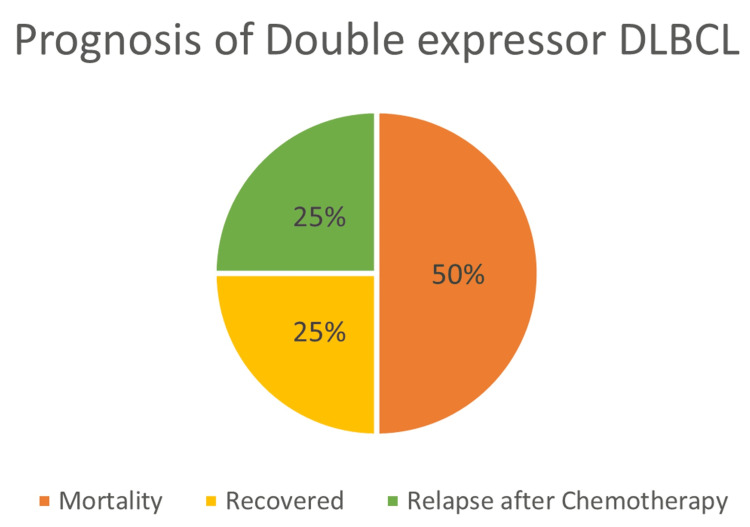
Prognostic statistics of DE DLBCL. DE: double expressor; DLBCL: diffuse large B-cell lymphoma.

Among the 11 individuals having double expressor subtype in the study, 6 (50%) passed away within six months, three participants (25%) managed to recover, while three individuals (25%) experienced a relapse after undergoing chemotherapy (Table 3, Figure 4).

**Table 3:** Prognosis of double expressor DLBCL on study population (n=11). DLBCL: diffuse large B-cell lymphoma.

Prognosis	Frequency	Percentage
Death within six months	6	50
Recovered	3	25
Relapse after chemotherapy	3	25
Total	11	100

Prognostic statistics of diffuse large B-cell lymphoma 

**Figure 5:**
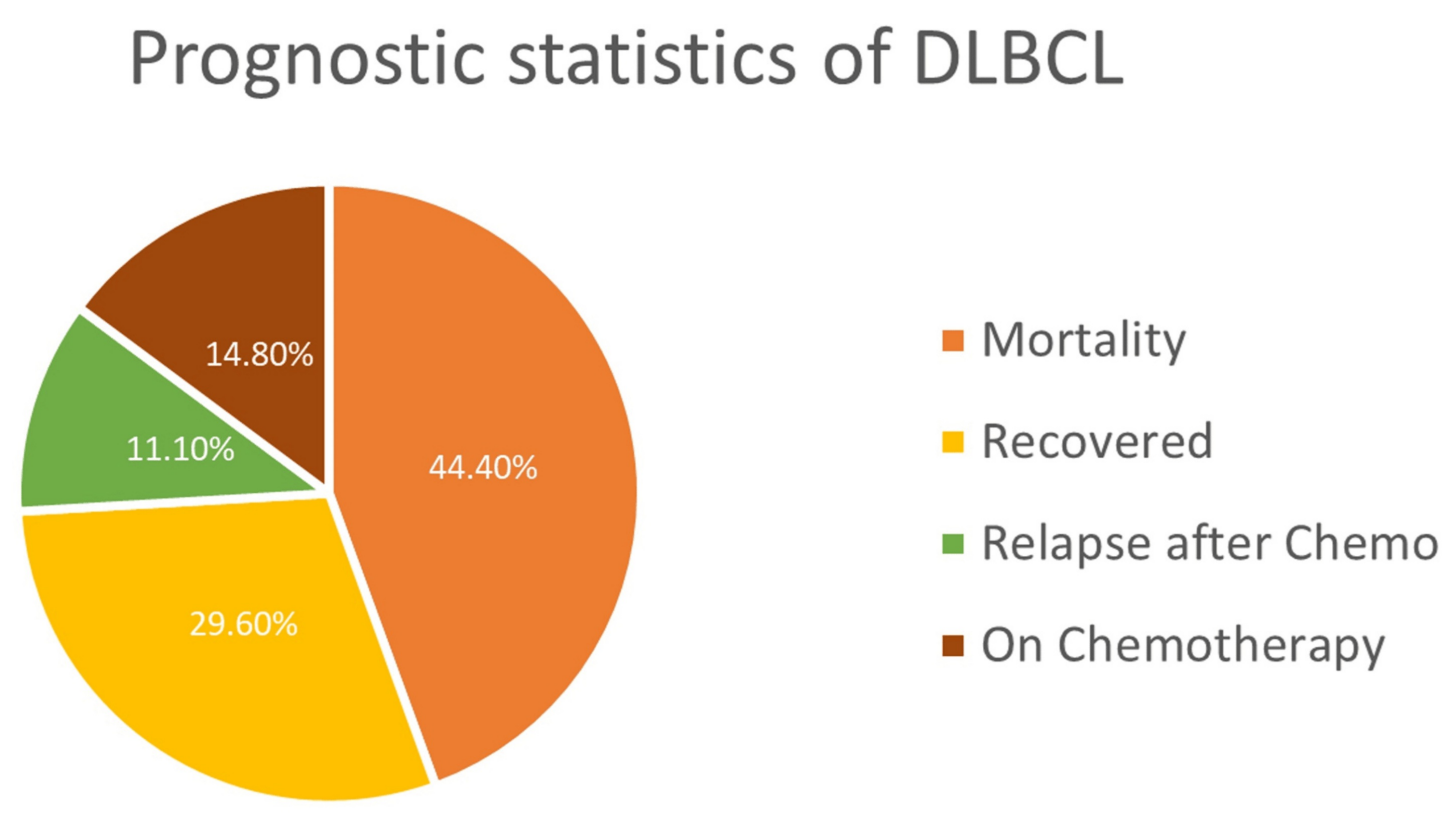
Prognostic statistics of DLBCL. DLBCL: diffuse large B-cell lymphoma.

**Figure 6:**
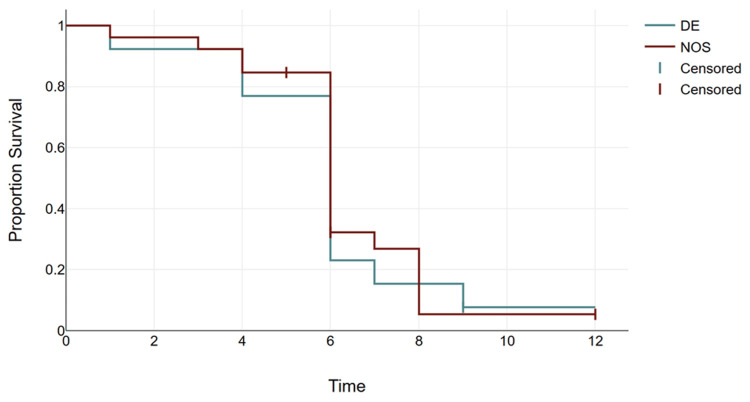
Proportion survival graph. DE: double expressor; NOS: not otherwise specified.

In a study population of 27 participants, 12 individuals (44.4%) succumbed within six months, while eight participants (29.6%) achieved recovery. A total of four participants (14.8%) remained on chemotherapy. Additionally, there were three cases (11.1%) of relapse (Table 4, Figure 5). The proportional survival graph shows a steep decline in survival for double expressors (Figure 6).

**Table 4:** Prognostic statistics of the study population (n=27).

Prognosis	Frequency	Percentage
Mortality within six months	12	44.4
Recovered	8	29.6
On chemotherapy	4	14.8
Relapse after chemotherapy	3	11.1
Total	27	100

DLBCL NOS is a type of medium to large neoplastic B cells arranged in a diffuse pattern with immunohistochemical staining showing membranous positivity for CD20, BCL6, BCL2, CD10, c-MYC, and negative for MUM-1 (Figures 7-14).

**Figure 7:**
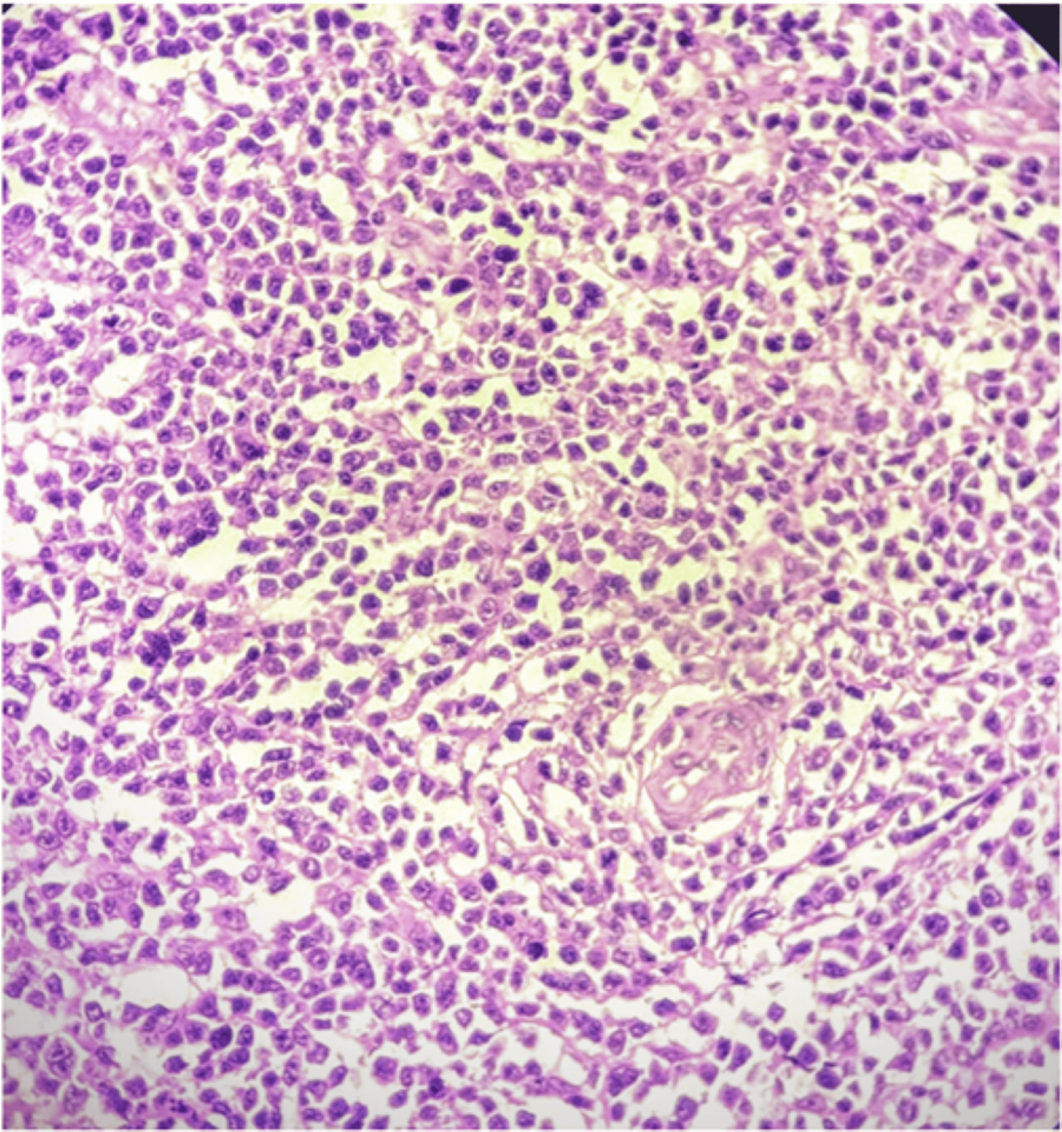
H&E (100x) section shows intermediate to large atypical lymphoid cells.

**Figure 8:**
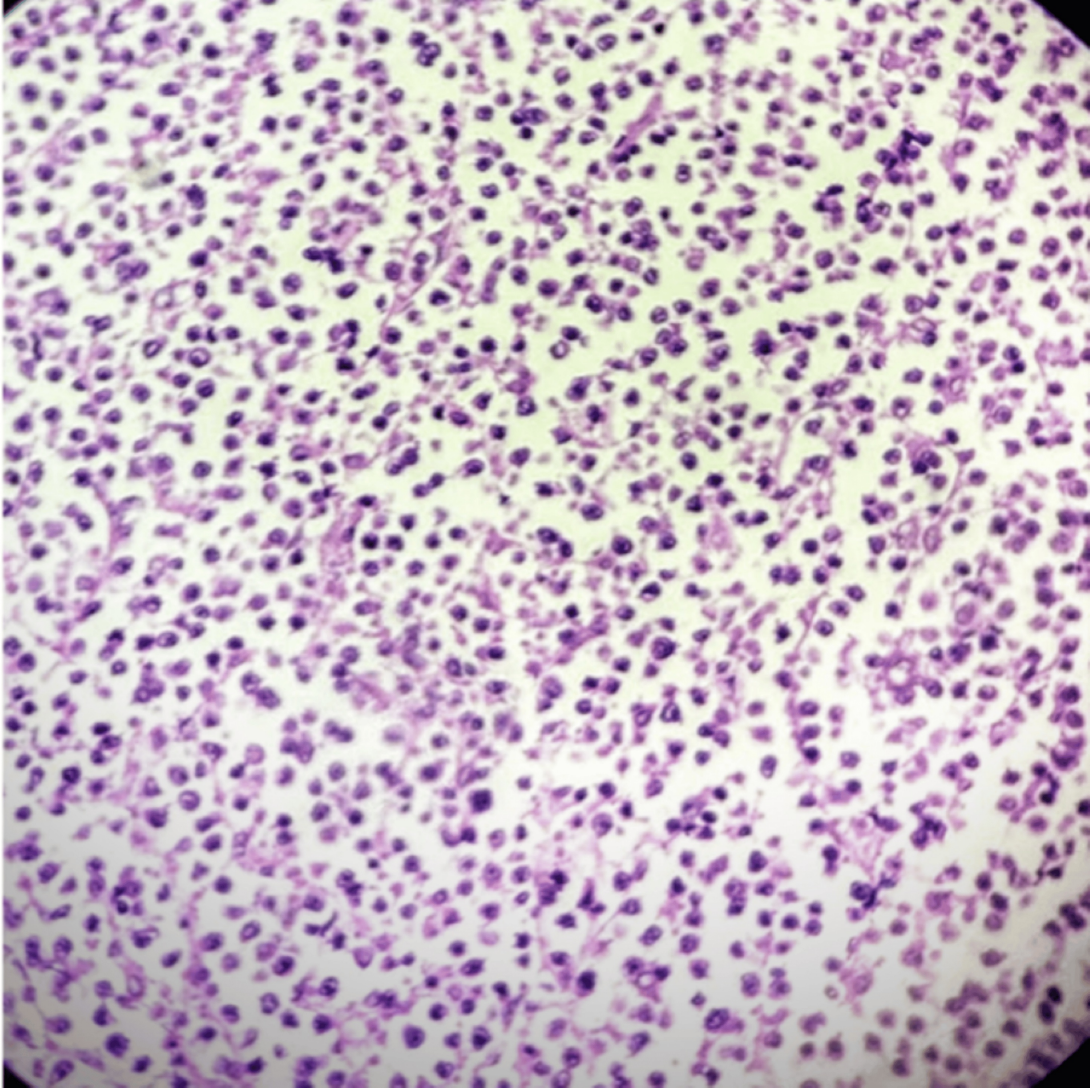
H&E (400x) section shows intermediate to large atypical lymphoid cells.

**Figure 9:**
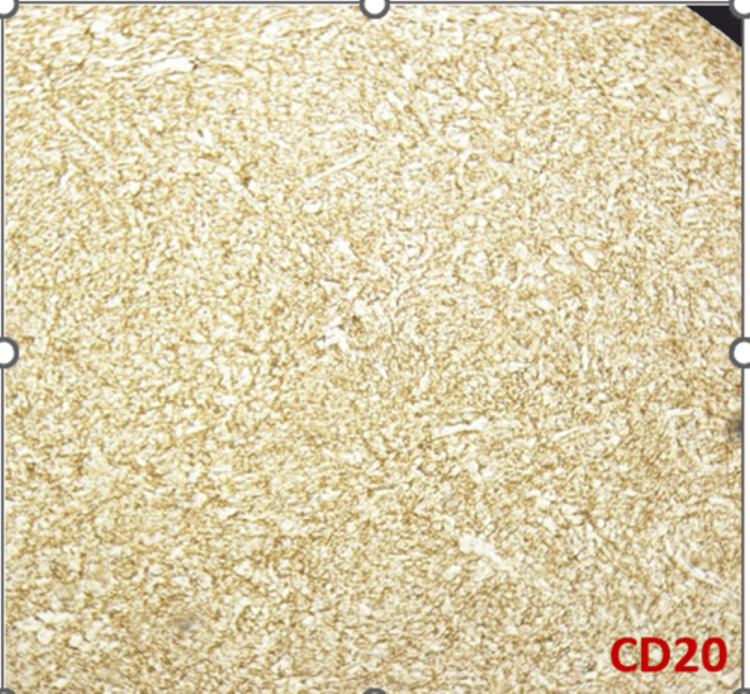
Immunohistochemical staining (100x) for CD20 shows diffuse membranous staining in neoplastic cells.

**Figure 10:**
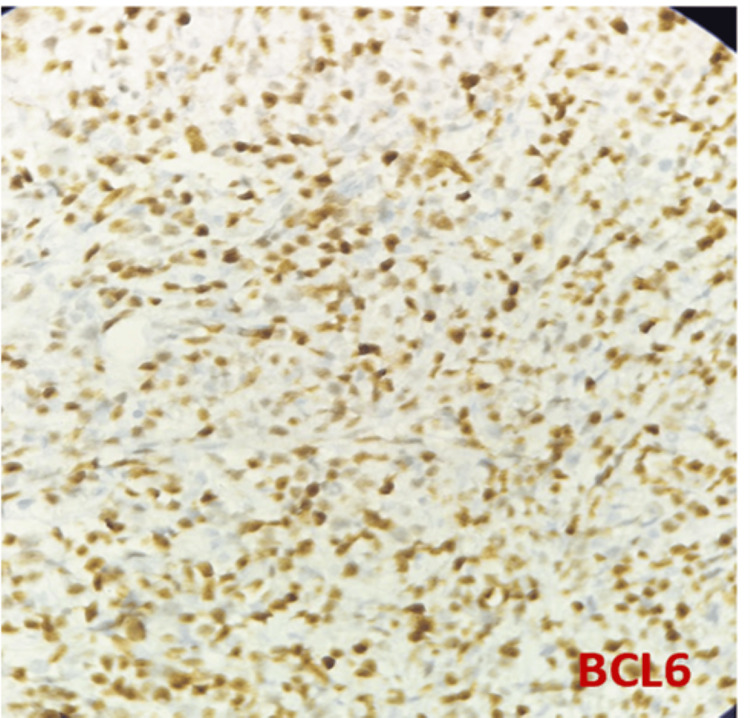
Immunohistochemical staining (400x) for BCL6 shows positivity in lymphoma cells.

**Figure 11:**
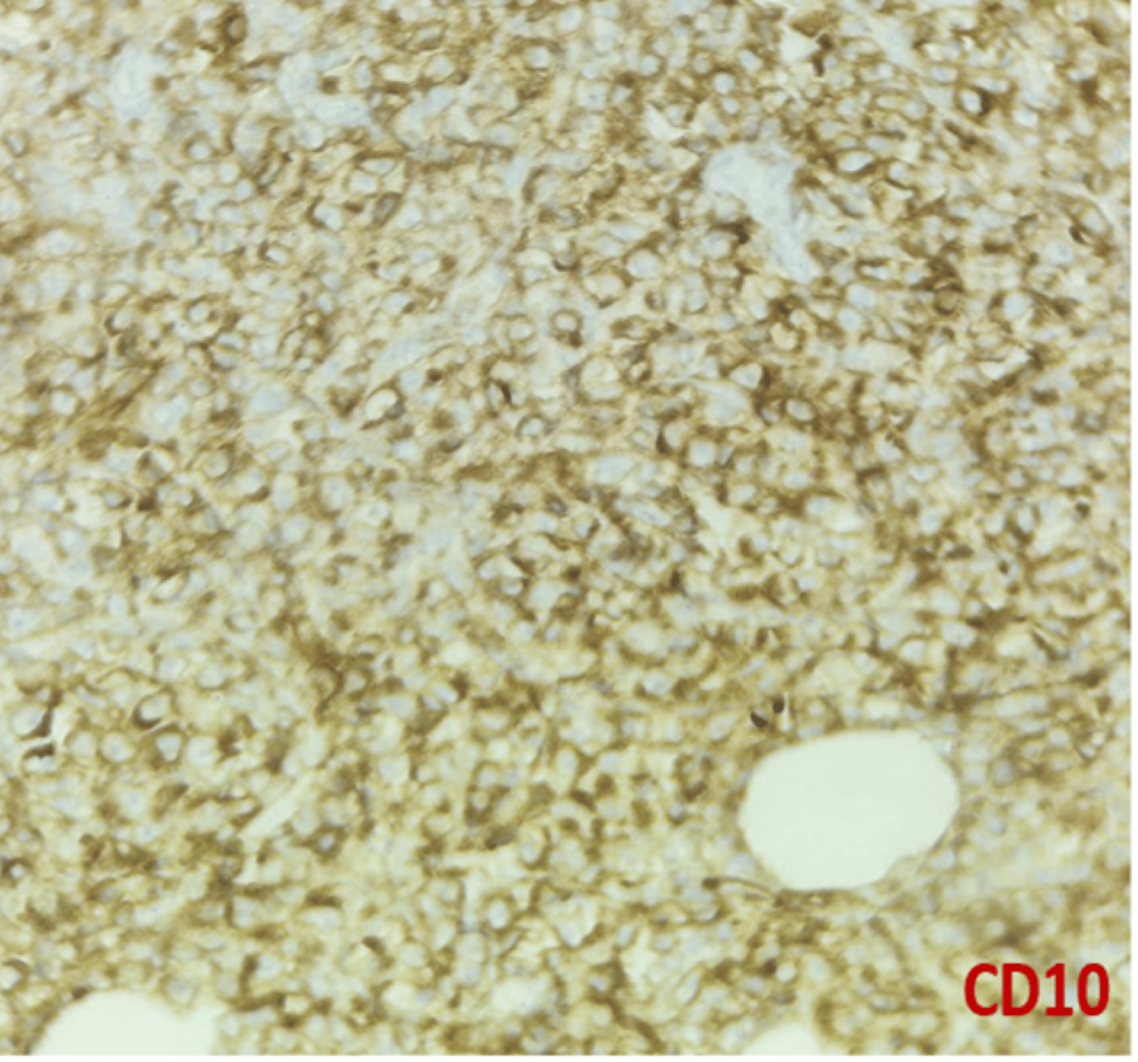
Immunohistochemical staining (400x) for CD10 shows membranous positivity in tumor cells.

**Figure 12:**
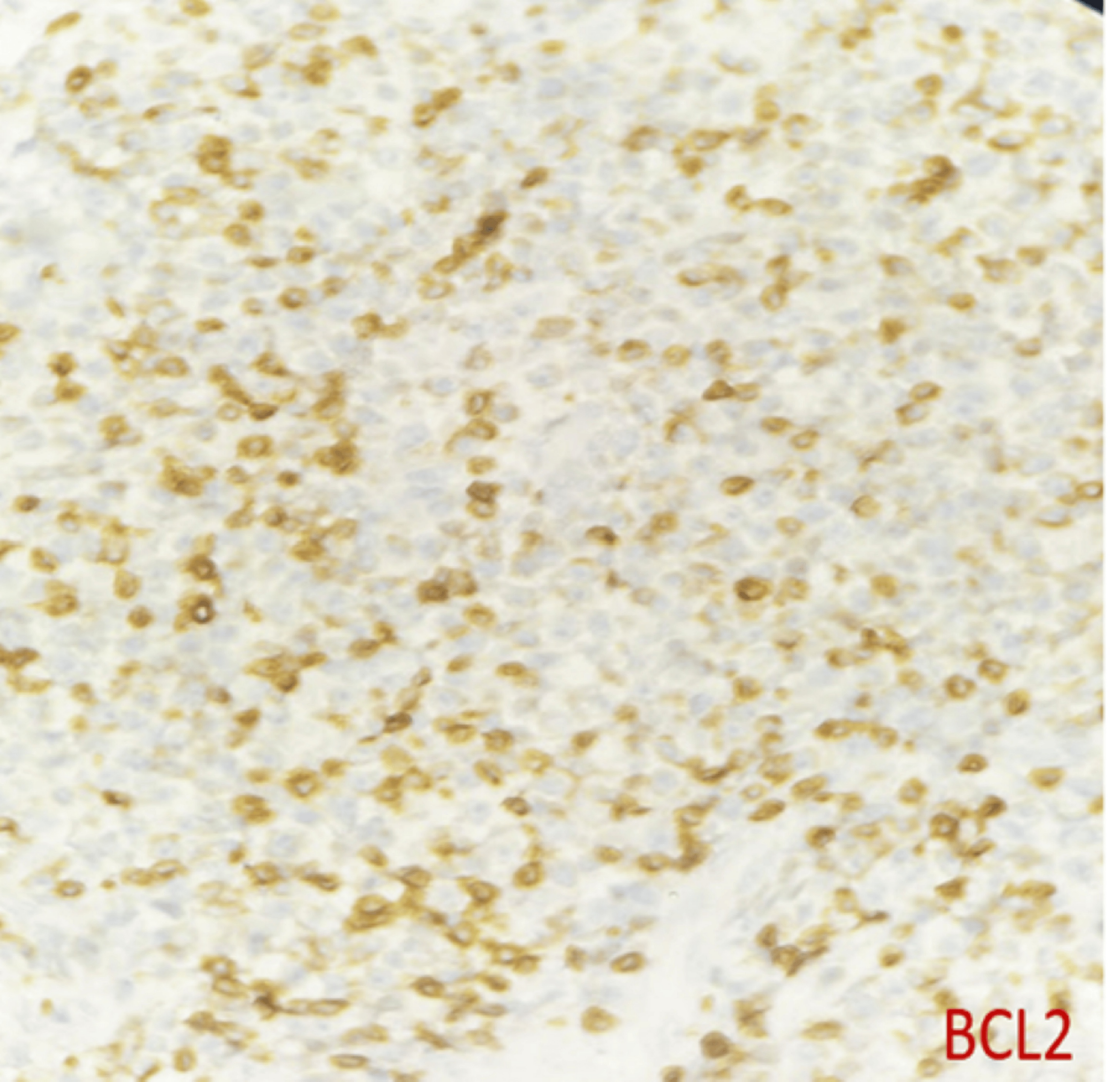
Immunohistochemical staining (400x) for BCL2 shows diffuse nuclear staining in tumor.

**Figure 13:**
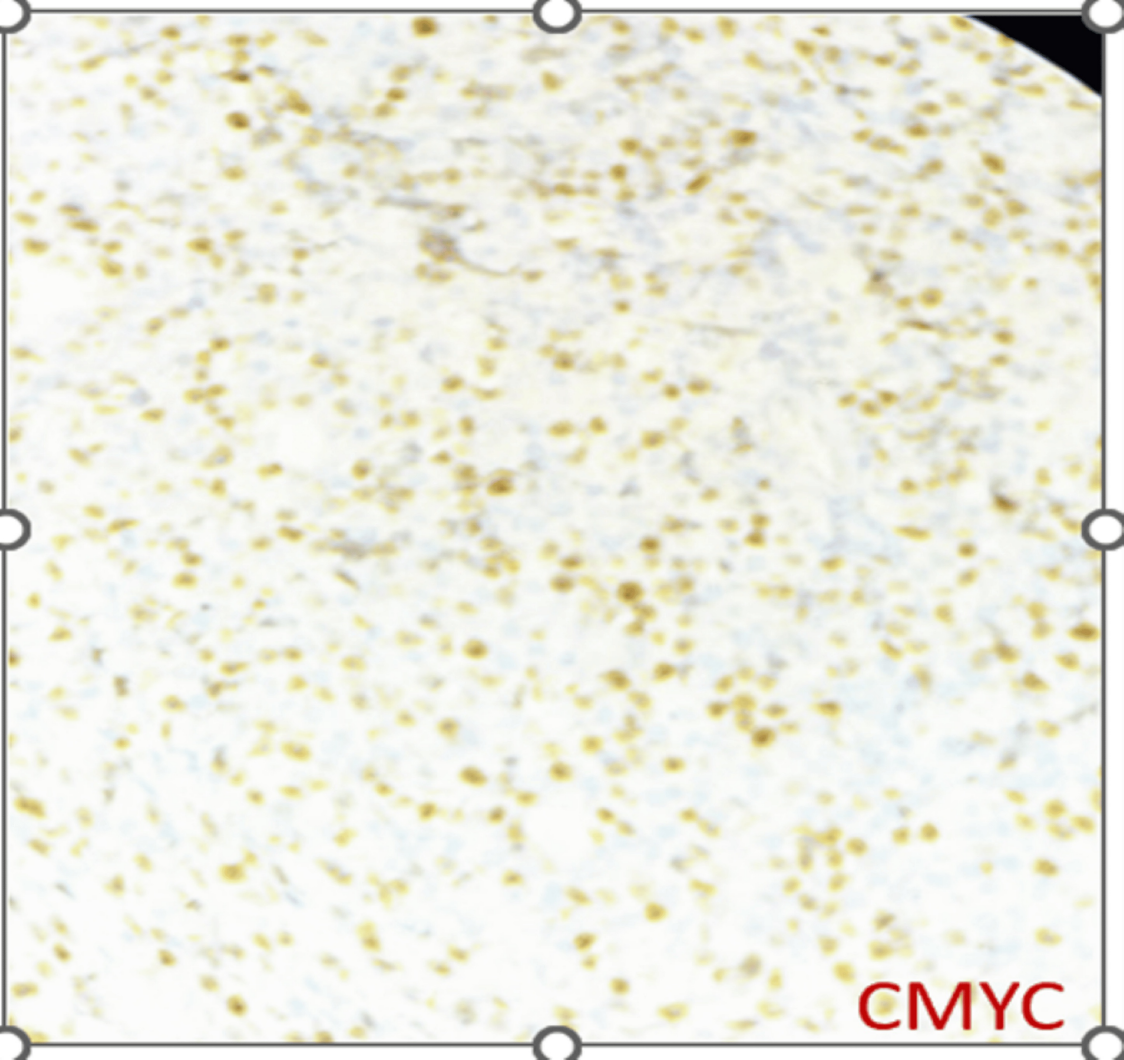
Immunohistochemical staining (100x) for c-MYC shows positive nuclear expression in tumor cells.

**Figure 14:**
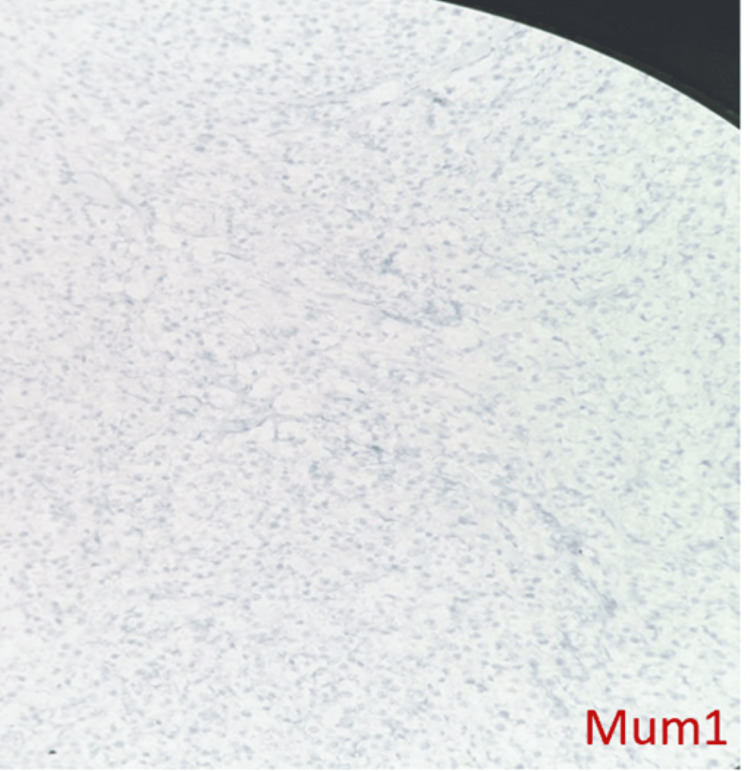
Immunohistochemical staining (100x) for MUM1 is negative.

## Discussion

Diffuse large B-cell lymphoma (DLBCL) NOS is a type of medium to large neoplastic B cells arranged in a diffuse pattern (Figures 7-14). In our cohort, 46.9% of cases exhibited co-expression of BCL2 and c-MYC, confirming a double expressor phenotype. This frequency is consistent with the findings of Riedell et al. [5], Asati et al. [6], who reported a similar prevalence in an Indian population. Minor discrepancies in reported frequencies across studies may stem from variations in antibody clone cut-offs and ethnic diversity [5,6].

Age distribution in our study revealed that 77.5% of DLBCL NOS cases occurred in individuals over 50 years (Figure 1), aligning with observations by Rungwittayatiwat et al., Hu et al., Hashmi et al., and Vijay et al. [7-10]. Among double expressor cases, 73.9% were above 50 years (Figure 2), reinforcing the age-related predilection noted in prior studies similar to Hashmi et al., at 70.2% [7,9,10].

Gender-wise, our findings showed a male predominance in both DLBCL NOS (63.3%) (Figure 1) and double expressor subtypes (57%) (Figure 2), which mirrors the gender distribution reported by Peña et al. [11]. This male bias may reflect underlying biological or environmental factors influencing disease susceptibility.

Cell-of-origin classification using the Hans algorithm revealed that 55.1% of DLBCL NOS cases were of the GCB subtype (Figure 1), whereas double expressor cases were more frequently non-GCB (52.2%) (Figure 2), corroborating findings by Rungwittayatiwat et al. [7], Hu et al. [8], Nagib et al. [12], and Abu Sabaa et al [13]. However, Nagib et al.'s study showed a frequency of 92.9% of double expressors expressing a non-GCB subtype.

Regarding the site of origin, nodal involvement was slightly more common (53.06%) in DLBCL NOS in our study, compared to (62.6%) in Shi et al [14], while 59.1% of double expressor cases were nodal (Figure 2), consistent with Ananthamurthy et al. [15], Mehta et al. [16], who highlighted nodal primacy in DLBCL presentations. The extranodal cases in our study, including vertebral, gastric, and adrenal sites, echo the anatomical diversity reported in the literature. This supports that double expressor status is more prevalent in the non-GCB subtype, which is often associated with poorer outcomes similar to Mehta et al. [16].

The Ki-67 proliferation index, assessed with an 80% cut-off, showed that only 18.4% of DLBCL NOS cases had high proliferative activity, compared to 35.3% in double expressor cases (Figure 3). However, in the Hashmi et al. study, the mean Ki-67 value of 72.94% of DLBCL NOS, this difference with our study might be due to the large sample size. There is elevated proliferation in double expressors, consistent with Huber et al. [17,18], who linked high Ki-67 with aggressive disease biology.

Retrospective follow-up of 27 cases revealed that 44.4% of patients with DLBCL died within six months, with double expressor patients showing a steeper decline in survival (50% mortality within six months) (Table 4, Figures 5, 6). Relapse rates were also higher among double expressors (25%) (Table 3, Figure 4), reinforcing the findings of Ip et al. [19] regarding treatment resistance and disease recurrence. These outcomes align with studies by Landsburg et al. [20,21], which underscore the poor prognosis associated with double expressor status.

Overall, our study reinforces the prognostic significance of the double expressor phenotype in DLBCL NOS, echoing global literature while contributing region-specific insights into clinicopathological correlations.

Limitations of study

Due to the limited sample size of 49 patients, while enough for initial correlation tests, it might not have enough statistical power to identify smaller or more subtle associations. The prognostic implications and survival outcomes for double expressor DLBCL could not be fully evaluated. Larger, multi-center studies are needed to validate these preliminary observations and to better understand the biological behavior and treatment responses in this subset of DLBCL. Such data will be essential for guiding risk-adapted therapy and improving patient outcomes.

The current study did not include molecular or genomic profiling, such as MYC rearrangements and BCL2 gene amplifications. Using techniques such as fluorescence in situ hybridization (FISH), PCR-based assays, and RNA sequencing could offer clearer molecular insights and help identify potential driver targets.

## Conclusions

This study highlights a significant prevalence of double expressor DLBCL, particularly among males and individuals over the age of 50, with a predominance of the non-germinal center B-cell (non-GCB) subtype and primary lymph node involvement. The identification of double expressor status marked by co-expression of c-MYC and BCL2, which holds important clinical value, as it is associated with more aggressive disease and poor prognosis. These findings emphasize the need for routine immunohistochemical screening for double expressors in DLBCL cases to facilitate early risk stratification. Recognizing this high-risk subgroup can aid clinicians in tailoring treatment strategies, potentially guiding the selection of more intensive or targeted therapies. Incorporating double expressor status into standard diagnostic protocols may ultimately contribute to more personalized and effective patient management.
